# A sporadic diffuse multiple cutaneous leiomyomatosis mimicking plexiform neurofibromatosis: A surgical challenge

**DOI:** 10.1002/ski2.414

**Published:** 2024-08-01

**Authors:** Gajanand M. Antakanavar, Bijaylaxmi Sahoo, Aneet Kaur, Ishu Ghiloria, Ajay Jangid, Bhavishya Shetty

**Affiliations:** ^1^ Department of Dermatology Maulana Azad Medical College New Delhi India; ^2^ Department of Pathology Maulana Azad Medical College New Delhi India; ^3^ Department of Surgery Maulana Azad Medical College New Delhi India

## Abstract

A 32‐year‐old male presented with diffuse plaques accompanied by multiple large, painful swellings predominantly distributed over the trunk, face and both upper and lower extremities for 11 years. There was no family history of similar complaints. The histopathological examination (HPE), supplemented with special stains confirmed the diagnosis of cutaneous leiomyoma (CL). The immunohistochemical analysis showed a strong expression of smooth muscle actin. The final diagnosis of sporadic diffuse multiple cutaneous leiomyomatosis was made. This presentation of CL is uncommon and resembled plexiform neurofibroma. The clinical diagnosis of this presentation was challenging. Therefore, HPE combined with special stains aided in confirming this surgically challenging rare tumour. In resource‐poor settings regular screening is required for early detection of renal cancer and other associated complications.

## Graphical abstract

Diffuse multiple cutaneous leiomyomatosis is rare entity with varied presentation. The present case had diffuse involvement of entire back, face with large tumour mimicking plexiform neurofibroma. This condition presented a potential challenge for both diagnosis and treatment.

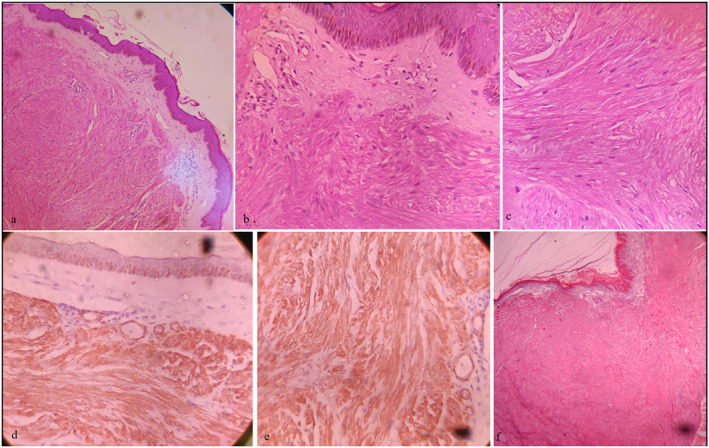

## 1 INTRODUCTION

Cutaneous leiomyoma (CL) arises from the arrector pili muscle of hair follicles. CL comprises of approximately 5% of all leiomyoma.^1^ These painful tumours tend to occur more frequently in adults, typically in the fifth to sixth decade of life rather than in children.^2^ They can be either inherited or sporadic. Clinical presentation can range from solitary, multiple, segmental and zosteriform.^1^, ^3^, ^4^ Multiple generalized, large lesions with diffuse involvement are indeed rare in cases of CLs. Herein, we present an atypical case of CL.

## 2 CASE PRESENTATION

A 32‐year adult male presented with multiple painful swellings on his face, trunk, arms and legs for 11 years. The condition initially presented as a small pea‐sized nodule on the back, progressively enlarging in both size and number to eventually encompass the entire trunk and face. Over the course of 1–2 years, lesions gradually developed on both the upper and lower extremities. These lesions were associated with moderate to severe pain with exacerbation on exposure to cold, emotional stress and during winter season. This significantly impacted the patient's quality of life. He did not have a history of diabetes mellitus, tuberculosis, recurrent infections, renal or cardiac abnormalities, and had not undergone any previous treatment for the condition. The patient had neither systemic symptoms nor a family history of a similar illness.

**FIGURE 1:**
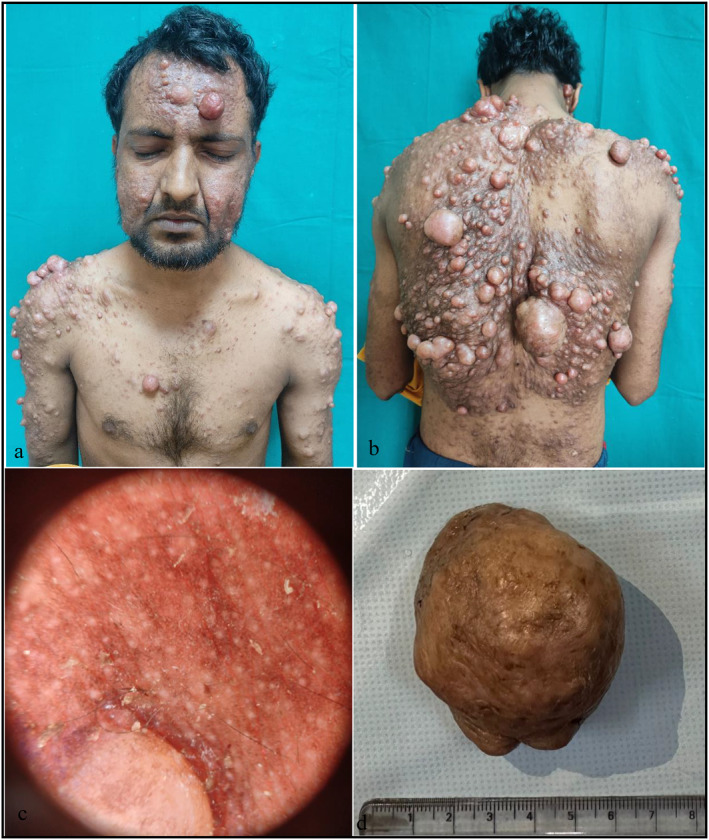
(a and b) There were multiple pink to reddish brown tender globular swellings and nodules of variable sizes present over face, trunk (a and b) and both extremities, with diffuse involvement of back in the form of indurated plaque (b). (c) Dermatoscopy revealed discrete brown reticular lines, along with numerous whitish clods of variable size and number. (d) The excised tumour measured 8 cm × 7 cm x 6 cm in size and exhibited shiny skin.

On examination, there was reddish‐brown thick indurated pendulous plaque at entire face and back (Figure 1a,b). There were multiple erythematous to skin‐coloured globular nodules as well as sessile to pedunculated tumours. These lesions were arising from the underlying thick plaque. The size of individual tumours varied from 1 × 1 cm (face) to as large as 8 × 7 cm (back) with smooth shiny surface, and few lesions showed tortuous telangiectasias. There was predominant involvement of the face and trunk (Figure 1a,b), with a few lesions distributed over bilateral shoulders, upper limbs and thighs. This presentation mimicked plexiform neurofibromatosis (NF). On palpation, the tumours were tender and firm in consistency. The button hole sign was negative. The ice cube test (contact with ice cube for 15 s) showed blanching followed by goose flesh elevation and pain at the site of contact.

Dermatoscopy revealed discrete brown reticular lines, accompanied by numerous whitish clods of variable size and number (Figure 1c). Based on the clinical examination, the patient was diagnosed with a differential diagnosis, including plexiform NF, CL, smooth muscle hamartoma, xanthomatous granuloma and adnexal tumour.

**FIGURE 2:**
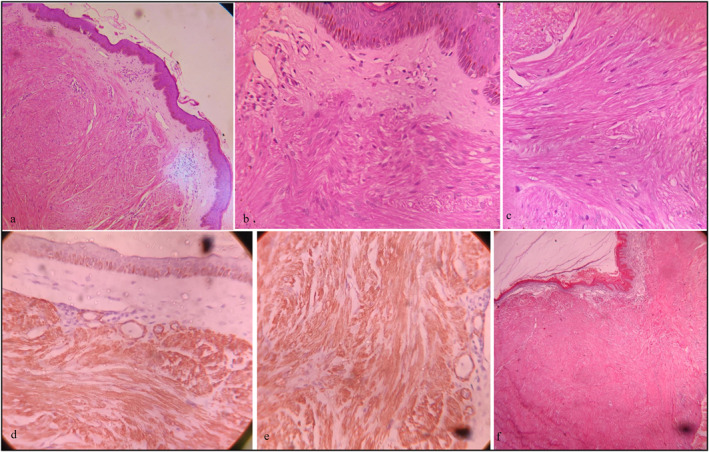
(a): Histopathological examination (Haematoxylin & Eosin stain, 100×) revealed minimal epidermal atrophy with increased in basal layer pigmentation. The papillary dermis (b) (400×) showed band of relative pale zone with few dilated capillaries, which was suggestive of Grenz zone. Both upper and deeper dermis showed (b and c) (400×) circumscribed, nonencapsulated tumour with interlacing fascicles with wavy muscle fibres. Individual spindle cells show (c) eosinophilic cytoplasm with elongated nuclei and blunt ends. There were no cytological atypia or mitosis. These features were consistent with benign cutaneous piloleiomyoma. (d and e) On immunohistochemistry (400×), cells were positive for smooth muscle actin. (f) Spindle cells and epidermis stained pink whereas adjacent collagen stained blue on Mason’s trichrome stain (100×).

On investigation, HIV serology was negative. The chest X‐ray showed multiple homogenous opacities in the thoracic region, likely originating from the soft tissue. The magnetic resonance imaging (MRI) of the chest, abdomen and pelvis revealed no abnormalities. HPE of excised giant tumour (Figure 1d) and biopsy from the indurated plaque on the back unveiled a circumscribed, non‐encapsulated tumour with a grenz zone beneath the epidermis (Figure 2a–c). The dermis comprised of interlacing fascicles with wavy muscle fibres. The individual spindle cells exhibited eosinophilic cytoplasm and elongated nuclei, with a subset demonstrating a perinuclear halo. No features suggestive of sarcoma, such as nuclear atypia, mitotic figures, haemorrhage or necrosis were observed. Immuno histochemistry was strongly positive for smooth muscle actin, favouring the presence of smooth muscles (Figure 2d,e). Masson's trichrome stain revealed that the smooth muscle cell cytoplasm stained red, whereas the collagenous fibrous tissue stained blue (Figure 2f). This presentation suggests a diagnosis of benign cutaneous piloleiomyoma. However, the lack of appropriate facilities rendered genetic screening for fumarate hydratase gene mutation unfeasible. The final diagnosis of sporadic diffuse multiple cutaneous piloleiomyoma without internal organ involvement was made.

The patient was started on Nifedipine 10 mg twice daily, Tramadol 50 mg twice daily and Gabapentin 300 mg once daily. The large lesion was excised (Figure 1d). This has notably alleviated pain, and there was significant improvement in the quality of life. The patient was advised for regular annual screening. Additionally, first‐degree blood relatives of patients were screened and advised for regular annual follow‐up.

## 3 DISCUSSION

CLs are rare benign painful tumours. They originate from the arrector pili muscle and its site of attachment. The trunk was commonly affected in multiple CL.^5^ However, the presentation can be either solitary, multiple or segmental.^1^, ^3^, ^4^, ^6^ The index case presented with diffuse plaque, as well as large sessile, and pedunculated globular swellings, reminiscent of plexiform NF. The size of CL varied from several millimetres to 4 cm.^7^ However, Goyal et al.,^8^ observed multiple plaques of size from 10 to 30 cm in diameter in a case of familial CL. An anecdotal report of hereditary leiomyoma mimicking NF was reported which had left T5 dermatomal involvement with few scattered lesions at extremities.^7^ The index had unusually larger sized (8 cm × 7 cm) lesions, coupled with extensive pendulous‐like involvement across the entire back and face. This unusual presentation is unique to this case.

Multiple piloleiomyomatosis can be associated with several conditions. These include Reed's syndrome, polycythaemia, as well as visceral involvement, notably affecting the gastrointestinal tract and retroperitoneal area. The few cases were also associated with multiple endocrine neoplasia type 1, rheumatoid arthritis, breast cancer, prostate cancer, bladder cancer, adrenal cortical adenoma and renal and ovarian cysts.^1^, ^9^, ^10^, ^11^ The most concerning association among them is Reed's syndrome, also known as hereditary leiomyomatosis and renal cell cancer syndrome (HLRCC). This arises from a mutation in the gene that encodes fumarate hydratase, located on chromosome 1q42.3.^1^ The most common manifestation of HLRCC include multiple cutaneous and uterine leiomyomas in 76%–100% of cases.^12^ The RCC was observed in 10%–16% of cases.^12^ Therefore, we have screened all family members clinically, and the results were unremarkable. Furthermore, the abdominal and pelvic MRI for the index case yielded normal findings. Mutation analysis could not be conducted due to inadequate facilities. Multiple CL with absence of systemic features had been reported.^1^ Hence, diagnosis of sporadic diffuse multiple cutaneous leiomyomatosis was made. In resource‐poor settings, clinical and radiological examination have become essential tools for screening. Type 2 papillary renal cell carcinoma is most common tumour associated with HLRCC, which tends to be hypoechoic on ultrasound and may be confused with a cyst.^11^ Therefore, renal ultra sonography is not recommended for screening. Hence renal CT (computed tomography) or MRI should be done either annually or once every 2 yearly for early detection of RCC. Consequently, it's recommended to perform annual screenings for both patients and their family members. The severe pain in these lesions could be because of local pressure exerted by the tumour on cutaneous nerves, infiltrating mast cells or muscle contraction on excitation via the sympathetic nervous system.^1^ Patients with such type of presentation are difficult to diagnose by clinical evaluation as they mimic other tumours. But the diagnosis can be established by HPE. Excising tumours of such extensive nature and successfully accomplishing grafting pose practical challenges. Therefore, the patient had been counselled regarding staged excision of painful tumours and the necessity for regular follow‐up.

In conclusion, the presence of multiple tumours, along with extensive pendulous involvement of the face and back resembles plexiform NF, which is an uncommon presentation. HPE with special stain is crucial in diagnosis such dilemmatic cases. The surgical management of extensive CL is challenging and further research is required for the management of such diffuse CL. Therefore, clinicians should be aware of such atypical presentations and conduct detailed investigations. Hence, regular follow‐up is crucial for early detection of systemic involvement, with the goal of reducing early mortality.

## CONFLICT OF INTEREST STATEMENT

None to declare.

## AUTHOR CONTRIBUTIONS

**Gajanand M. Antakanavar**: Conceptualization (lead); data curation (lead); formal analysis (lead); investigation (equal); methodology (equal); resources (lead); software (equal); validation (equal); visualization (equal); writing – original draft (lead); writing – review & editing (lead). **Bijaylaxmi Sahoo**: Formal analysis (equal); project administration (equal); supervision (equal); validation (equal); visualization (equal); writing – review & editing (equal). **Aneet Kaur**: Formal analysis (equal); validation (equal); visualization (equal); writing – review & editing (equal). **Ishu Ghiloria**: Conceptualization (equal); formal analysis (equal); methodology (equal); resources (equal); visualization (equal). **Ajay Jangid**: Data curation (equal); formal analysis (supporting); investigation (supporting); resources (supporting). **Bhavishya Shetty**: Formal analysis (equal); investigation (equal); supervision (supporting); visualization (equal).

## ETHICS STATEMENT

Not applicable.

## PATIENT CONSENT

Written patient consent for publication was obtained.

## Data Availability

The data underlying this article will be shared on reasonable request to the corresponding author.
